# Supplementary material for: External validation of the Meggitt-Wagner, Texas University, SINBAD, and Saint Elian classifications for predicting major amputation in patients with diabetes at a public hospital in Peru
Source: PLoS One. 2026 Jan 21;21(1):e0327601. doi: 10.1371/journal.pone.0327601 (PMC12822936; doi:10.1371/journal.pone.0327601)
Supplement: S2 Table — (DOCX) [file pone.0327601.s002.docx]

**S2 Table. TRIPOD Checklist for External Validation Studies**

| **Item** | **Recommendation** | **Page** |
| --- | --- | --- |
| 1 | Identify study as external validation in title/abstract | p. 1–2 |
| 2 | Provide structured summary of background, methods, results | p. 3 |
| 3 | Explain medical context, rationale for validation | p. 4 |
| 4 | Specify objectives of validation | p. 4 |
| 5a | Describe source of data, study design | p. 5 |
| 5b | Describe key dates, recruitment and follow-up | p. 5 |
| 6a | Describe eligibility criteria | p. 6 |
| 6b | Provide details of treatment received (if relevant) | p. 6 |
| 7a | Clearly define outcome and assessment method | p. 8 |
| 7b | Describe blinding of outcome assessment (if applicable) | p. 8 |
| 8 | Clearly define predictors and how measured | p. 7–8 |
| 9 | Describe sample size considerations | p. 10 |
| 10a | Describe how missing data were handled | p. 10 |
| 10b | Report any imputation methods (if applied) | p. 10 |
| 11 | Describe all statistical analysis methods | p. 11 |
| 12 | Report participant flow and characteristics | p. 12 |
| 13a | Report validation performance measures (AUC, CI) | p. 17 |
| 13b | Compare performance across subgroups | p. 17-8 |
| 14 | Discuss limitations of validation | p. 24-25 |
| 15 | Discuss clinical implications and interpretation | p. 24-25 |
| 16 | Funding, conflicts of interest | p. Yes |
