# Supplementary material for: External validation of the Meggitt-Wagner, Texas University, SINBAD, and Saint Elian classifications for predicting major amputation in patients with diabetes at a public hospital in Peru
Source: PLoS One. 2026 Jan 21;21(1):e0327601. doi: 10.1371/journal.pone.0327601 (PMC12822936; doi:10.1371/journal.pone.0327601)
Supplement: S3 Table — (DOCX) [file pone.0327601.s003.docx]

**S3 Table. Missing data by variable (n = 342)**

| Variable | Non-missing (n) | Missing (n) | Missing (%) |
| --- | --- | --- | --- |
| Age | 342 | 0 | 0.0% |
| Sex | 342 | 0 | 0.0% |
| Educational level | 272 | 70 | 20.5% |
| Body mass index | 244 | 98 | 28.7% |
| Duration of diabetes | 303 | 39 | 11.4% |
| Previous diabetes treatment | 315 | 27 | 7.9% |
| Estimated glomerular filtration rate (eGFR) | 283 | 59 | 17.3% |
| Hypertension | 340 | 2 | 0.6% |
| Coronary artery disease | 323 | 19 | 5.6% |
| Stroke history | 313 | 29 | 8.5% |
| Previous major amputation | 313 | 29 | 8.5% |
| Ulcer type (new or recurrent) | 336 | 6 | 1.8% |
| Ulcer extension | 342 | 0 | 0.0% |
| Ulcer depth | 342 | 0 | 0.0% |
| Ulcer location | 342 | 0 | 0.0% |
| Ischemia (Doppler waveform) | 342 | 0 | 0.0% |
| Infection severity (IDSA classification) | 342 | 0 | 0.0% |
| HbA1c (%) | 239 | 103 | 30.1% |
| LDL cholesterol | 198 | 144 | 42.1% |
| Hemoglobin | 342 | 0 | 0.0% |
| Serum albumin | 124 | 218 | 63.7% |
| Hospitalization status | 342 | 0 | 0.0% |
| Length of hospital stay | 342 | 0 | 0.0% |
| Meggitt–Wagner classification | 342 | 0 | 0.0% |
| Texas University classification (all components) | 342 | 0 | 0.0% |
| SINBAD score | 342 | 0 | 0.0% |
| Saint Elian score | 342 | 0 | 0.0% |
| Outcome: major amputation | 342 | 0 | 0.0% |
