# Supplementary material for: External validation of the Meggitt-Wagner, Texas University, SINBAD, and Saint Elian classifications for predicting major amputation in patients with diabetes at a public hospital in Peru
Source: PLoS One. 2026 Jan 21;21(1):e0327601. doi: 10.1371/journal.pone.0327601 (PMC12822936; doi:10.1371/journal.pone.0327601)
Supplement: S4 Table — (DOCX) [file pone.0327601.s004.docx]

**S4 Table.** Incidence of major amputation across detailed categories of diabetic foot classification systems.

|  | Major amputation | No major amputation | p |
| --- | --- | --- | --- |
| Meggitt -Wagner |  |  |  |
| 1 | 0 (0.0) | 125 (100.0) | <0.001 ^d^ |
| 2 | 0 (0.0) | 58 (100.0) |  |
| 3 | 3 (27.3) | 8 (72.7) |  |
| 4 | 35 (23.8) | 112 (76.2) |  |
| 5 | 1 (100.0) | 0 (0.0) |  |
| U. Texas |  |  |  |
| Depth |  |  |  |
| 1 | 0 (0.0) | 125 (100.0) | <0.001 ^d^ |
| 2 | 0 (0.0) | 58 (100.0) |  |
| 3 | 39 (24.5) | 120 (75.5) |  |
| Ischemia | 34 (16.7) | 170 (83.3) | <0.001^a^ |
| Infection | 39 (15.5) | 212 (84.5) | <0.001 ^a^ |
| Categories |  |  |  |
| 1a | 0 (0.0) | 49 (100.0) | <0.001 ^b^ |
| 1b | 0 (0.0) | 16 (100.0) |  |
| 1c | 0 (0.0) | 34 (100.0) |  |
| 1d | 0 (0.0) | 26 (100.0) |  |
| 2a | 0 (0.0) | 3 (100.0) |  |
| 2b | 0 (0.0) | 22 (100.0) |  |
| 2c | 0 (0.0) | 3 (100.0) |  |
| 2d | 0 (0.0) | 30 (100.0) |  |
| 3ª | 0 (0.0) | 1 (100.0) |  |
| 3b | 5 (10.6) | 42 (89.4) |  |
| 3c | 0 (0.0) | 1 (100.0) |  |
| 3d | 34 (30.9) | 76 (69.1) |  |
| SINBAD* |  |  |  |
| Site midfoot and hindfoot | 9 (10.3) | 103 (89.8) | 0.680 ^a^ |
| Ischemia | 34 (16.7) | 170 (83.3) | <0.001 ^a^ |
| Neuropathy | 38 (12.6) | 264 (87.4) | 0.060 ^a^ |
| Infection | 39 (15.6) | 212 (84.5) | <0.001 ^a^ |
| Area > 1cm | 39 (13.1) | 258 (86.9) | 0.010 ^a^ |
| Depth | 39 (18.0) | 178 (82.0) | <0.001 |
| Number of components |  |  |  |
| 0 | 0 (0.0) | 9 (100.0) | 0.001 ^b^ |
| 1 | 0 (0.0) | 20 (100.0) |  |
| 2 | 0 (0.0) | 35 (100.0) |  |
| 3 | 0 (0.0) | 44 (100.0) |  |
| 4 | 5 (6.8) | 69 (93.2) |  |
| 5 | 26 (21.7) | 94 (78.3) |  |
| 6 | 8 (20.0) | 32 (80.0) |  |
| Median [RIC] | 5 [5-5] | 4 [3-5] | <0.001 ^d^ |
| Saint Elian |  |  |  |
| Categories |  |  |  |
| 0-5 | 0 (0.0) | 6 (100.0) | <0.001 ^d^ |
| 6-10 | 0 (0.0) | 87 (100.0) |  |
| 11-15 | 3 (2.7) | 107 (97.3) |  |
| 16-20 | 16 (16.5) | 81 (83.5) |  |
| 21-25 | 20(47.6) | 22 (52.4) |  |
| Median [IQR] | 19 [19-22] | 14 [9-17] |  |

Values are presented as n (%). Statistical tests: a Chi-square test; b Fisher’s exact test; c Student’s t-test; d Mann–Whitney U test. The SINBAD classification did not include the “absent” category, which was omitted in this analysis. IQR: Interquartile range.
