# Supplementary material for: External validation of the Meggitt-Wagner, Texas University, SINBAD, and Saint Elian classifications for predicting major amputation in patients with diabetes at a public hospital in Peru
Source: PLoS One. 2026 Jan 21;21(1):e0327601. doi: 10.1371/journal.pone.0327601 (PMC12822936; doi:10.1371/journal.pone.0327601)
Supplement: S5 Table — (DOCX) [file pone.0327601.s005.docx]

**S5 Table.** Statistical power of pairwise comparisons between ROC curves of diabetic foot classifications

|  | Saint Elian | SINBAD | Texas University 3D |
| --- | --- | --- | --- |
| Meggitt-Wagner | 99% | 71.4% | 18.7% |
| SINBAD | 99.9% | ------ | 88.7% |
| Texas University 3D | 78.4% | ------- | ------ |

Power values are expressed as percentages. Calculations assumed 39 cases and 303 controls, with a two-sided α = 0.05. AUROC values were taken from the main analysis (Figure 1). Dashes indicate comparisons that were not applicable
