# Supplementary material for: External validation of the Meggitt-Wagner, Texas University, SINBAD, and Saint Elian classifications for predicting major amputation in patients with diabetes at a public hospital in Peru
Source: PLoS One. 2026 Jan 21;21(1):e0327601. doi: 10.1371/journal.pone.0327601 (PMC12822936; doi:10.1371/journal.pone.0327601)
Supplement: S6 Table — (DOCX) [file pone.0327601.s006.docx]

**S6 Table. Prognostic discriminative capacity across the full range of categories and cut-off points of diabetic foot classifications for major amputation.**

|  | Sensitivity (95% CI) | Specificity (95% CI) | PPV (95% CI) | NPV (95% CI) | LR [+] (95% CI) | LR [-] (95% CI) | Youden index |
| --- | --- | --- | --- | --- | --- | --- | --- |
| Meggitt-Wagner |  |  |  |  |  |  |  |
| ≥ 2 | 100.0 (91-100) | 41.3 (35.7-47) | 17.9 (13.1-23.7) | 100.0 (97.1-100) | 1.70 (1.55-1.87) | 0.00 | 41.3 |
| ≥ 3 | **100.0 (91-100)** | **60.4 (54.6-65.9)** | **24.5 (18.1-32)** | **100.0 (98-100)** | **2.52 (2.2-2.9)** | **0.00** | **60.4** |
| ≥ 4 | 92.3 (79.1-98.4) | 63.0 (57.3-68.5) | 24.3 (17.7-32.1) | 98.5 (95.5-99.7) | 2.49 (2.1-2.97) | 0.12 (0.041-0.363) | 55.3 |
| ≥ 5 | 2.6 (0.64-13.6) | 100.0 (98.8-100) | 100.0 (2.5-100) | 88.9 (85-92) | --- | 0.97 (0.92-1.03) | 2.5 |
| TU Depth |  |  |  |  |  |  |  |
| ≥ 2 | 100 (91-100) | 41.3 (35.7-47) | 17.9 (13.1-23.7) | 100.0 (97.1-100) | 1.70 (1.55-1.87) | 0.00 | 41.3 |
| ≥ 3 | **100 (91-100)** | **60.4 (54.6-65.9)** | **24.5 (18.1-32)** | **100.0 (98-100)** | **2.52 (2.2-2.9)** | **0.00** | **60.4** |
| TU Ischemia |  |  |  |  |  |  |  |
| Yes | 87.2 (72.6-95.7) | 43.9 (38.2-49.7) | 16.6 (11.8-22.5) | 96.4 (91.7-98.8) | 1.55 (1.33-1.82) | 0.29 (0.12-0.66) | 31.1 |
| TU infection |  |  |  |  |  |  |  |
| Yes | 100.0 (91-100) | 30.0 (24.9-35.5) | 15.5 (11.3-20.6) | 100.0 (96-100) | 1.43 (1.33-1.54) | 0.00 | 30.0 |
| TU 3D |  |  |  |  |  |  |  |
| 3D | **87.2 (72.6-95.7)** | **74.9 (69.6-79.7)** | **30.9 (22.4-40.4)** | **97.8 (95-99.3)** | **3.47 (2.76-4.37)** | **0.17 (0.07-0.38)** | **62.1** |
| SINBAD |  |  |  |  |  |  |  |
| ≥ 1 | 100.0 (91-100) | 2.9 (1.37-5.56) | 11.7 (8.46-15.7) | 100.0 (66.4-100) | 1.03 (1.01-1.05) | 0.00 | 2.9 |
| ≥ 2 | 100.0 (91-100) | 9.6 (6.5-13.5) | 12.4 (9.01-16.6) | 100.0 (88.1-100) | 1.11 (1.07-1.15) | 0.00 | 9.6 |
| ≥ 3 | 100.0 (91-100) | 21.1 (16.7-26.2) | 14.0 (10.2-18.7) | 100.0 (94.4-100) | 1.27 (1.2-1.34) | 0.00 | 21.1 |
| ≥ 4 | 100.0 (91-100) | 35.6 (30.2-41.3) | 16.7 (12.1-22.1) | 100.0 (96.6-100) | 1.55 (1.43-1.69) | 0.00 | 35.6 |
| ≥ 5 | **87.2 (72.6-95.7)** | **58.4 (52.6-64)** | **21.3 (15.2-28.4)** | **97.3 (93.7-99.1)** | **2.09 (1.75-2.51)** | **0.21 (0.09-0.5)** | **45.6** |
| ≥ 6 | 20.5 (9.3-36.5) | 89.4 (85.4-92.7) | 20.0 (9.05-35.6) | 89.7 (85.7-92.9) | 1.94 (0.96-3.91) | 0.89 (0.75-1.05) | 9.9 |
| Saint Elian |  |  |  |  |  |  |  |
| ≥ 6 | 100.0 (91-100) | 1.98 (0.73-4.26) | 11.6 (8.39-15.5) | 100.0 (54.1-100) | 1.02 (1-1.04) | 0.00 | 1.98 |
| ≥ 7 | 100.0 (91-100) | 10.6 (7.3-14.6) | 12.6 (9.1-16.8) | 100.0 (89.1-100) | 1.12 (1.08-1.16) | 0.00 | 10.6 |
| ≥ 8 | 100.0 (91-100 | 17.8 (13.7-22.6) | 13.5 (9.91-18) | 100.0 (93.4-100) | 1.22 (1.15-1.28) | 0.00 | 17.8 |
| ≥ 9 | 100.0 (91-100 | 22.1 (17.6-27.2) | 14.2 (10.3-18.9) | 100.(94.6-100) | 1.28 (1.21-1.36) | 0.00 | 22.1 |
| ≥ 10 | 100.0 (91-100 | 22.1 (17.6-27.2) | 15 (10.3-18.9) | 100.0 (95.6-100) | 1.37 (1.28-1.47) | 0.00 | 27.1 |
| ≥ 11 | 100.0 (91-100 | 30.7 (25.5-36.2) | 15.7 (11.4-20.8) | 100.0 (96.1-100) | 1.44 (1.34-1.56) | 0.00 | 30.7 |
| ≥ 12 | 100.0 (91-100 | 36 (30.6-41.7) | 16.7 (12.2-22.2) | 100.0 (96.7-100) | 1.56 (1.44--1.7) | 0.00 | 35.9 |
| ≥ 13 | 100.0 (91-100 | 42.9 (37.3-48.7) | 18.4 (13.4-24.3) | 100.0 (97.2-100) | 1.75 (1.59-1.93) | 0.00 | 42.9 |
| ≥ 14 | 97.4 (86.5-99.9) | 49.8 (44.1-55.6) | 20 (14.6-26.4) | 100.0 (96.4-100) | 1.94 (1.72-2-2) | 0.05 (0.07-0.35) | 47.3 |
| ≥ 15 | 97.4 (86.5-99.9) | 59.1 (53.3-64.7) | 23.5 (17.2-30.7) | 99.4 (96.8-100) | 2.38 (2.06-2.75) | 0.04 (0.06-0.30) | 56.5 |
| ≥ 16 | 92.3 (79.1-98.4) | 66.0 (60.4-71.3) | 25.9 (18.8-34) | 98.5  (95.7-99.7) | 2.72 (2.27-3.25) | 0.12 (0.03-0.34) | 58.3 |
| ≥ 17 | 89.7 (75.8-97.1) | 74.3 (68.9-79.1) | 31.0 (22.6-40-4) | 98.3 (95.6-99.5) | 3.48 (2.8-4.3) | 0.13 (0.05-0.35) | 64.0 |
| ≥ 18 | **87.2 (72.6-95.7)** | **82.2 (77.4-86.4)** | **38.6 (28.4-49.6)** | **98.0 (95.5-99.4)** | **4.89 (3.73-6.41)** | **0.15 (0.68-0.35)** | **69.4** |
| ≥ 19 | 76.9 (60.7-88.9) | 88.1 (83.9-91.5) | 45.5 (33.1-58.2) | 96.7 (93.7-98.5) | 6.47 (4.56-9.2) | 0.26 (0.14-0.46) | 65.0 |
| ≥ 20 | 71.8 (55.1-85) | 90.1 (86.2-93.2) | 48.3 (35-61.8) | 96.1 (93.2-98.1) | 7.25 (4.9-10.7) | 0.31 (0.18-0.51) | 61.9 |
| ≥ 21 | 51.3 (34.8-67.6) | 92.7 (89.2-95.4) | 47.6 (32-63.6) | 93.7 (90.3-96.1) | 7.06 (4.26-11.7) | 0.52 (0.38-0.72) | 44.0 |
| ≥ 22 | 38.5 (23.4-55.4) | 96.0 (93.2-97.9) | 55.6 (35.3-74.5) | 92.4 (88.9-95.1) | 9.71 (4.91-19.2) | 0.64 (0.49-0.82) | 34.5 |
| ≥ 23 | 15.4 (5.86-30.5) | 98.0 (95.7-99.3) | 50.0 (21.1-78.9) | 90.0 (86.2-93) | 7.76 (2.63-22.9) | 0.86 (0.75-0.98) | 13.4 |
| ≥ 24 | 7.7 (1.62-20.9) | 99.7 (98.2-100) | 75.0 (19.4-99.4) | 89.3 (85.6-92.4) | 23.3 (2.49-219) | 0.92 (0.84-1.01) | 7.4 |

PPV: Positive predictive value. NPV: Negative predictive value. LR(+): Positive likelihood ratio. LR(–): Negative likelihood ratio.

95% CI in parentheses. Youden index = sensitivity + specificity – 1. A dash (---) indicates not applicable.
