# Supplementary material for: External validation of the Meggitt-Wagner, Texas University, SINBAD, and Saint Elian classifications for predicting major amputation in patients with diabetes at a public hospital in Peru
Source: PLoS One. 2026 Jan 21;21(1):e0327601. doi: 10.1371/journal.pone.0327601 (PMC12822936; doi:10.1371/journal.pone.0327601)
Supplement: S7 Table — (DOCX) [file pone.0327601.s007.docx]

**S7 Table. AUROC (95% CI) of each classification system for predicting any amputation, overall and stratified by hospitalization status**

| Classification system | All patients (n=342) | Hospitalized (n=109) | Not hospitalized (n=233) |
| --- | --- | --- | --- |
| Meggitt–Wagner | 0.8606 (0.8183–0.8947) | 0.6678 (0.5731–0.7568) | 0.8718 (0.8213–0.9114) |
| Saint Elian | 0.8430 (0.7991–0.8791) | 0.6564 (0.5636–0.7485) | 0.8163 (0.7596–0.8631) |
| SINBAD | 0.7023 (0.6502–0.7498) | 0.5146 (0.4161–0.6106) | 0.6736 (0.6096–0.7336) |
| UT: all vs 3D | 0.7686 (0.7207–0.8126) | 0.6889 (0.5923–0.7734) | 0.6936 (0.6318–0.7537) |
| UT: depth | 0.8587 (0.8183–0.8947) | 0.6676 (0.5731–0.7568) | 0.8709 (0.8213–0.9114) |
| UT: ischemia | 0.5878 (0.5335–0.6404) | 0.6193 (0.5260–0.7148) | 0.4975 (0.4319–0.5639) |
| UT: infection | 0.6873 (0.6351–0.7359) | 0.5139 (0.4161–0.6106) | 0.7066 (0.6452–0.7657) |

AUC = area under the ROC curve; CI = confidence interval. Values estimated using binomial exact method.
