# Supplementary material for: External validation of the Meggitt-Wagner, Texas University, SINBAD, and Saint Elian classifications for predicting major amputation in patients with diabetes at a public hospital in Peru
Source: PLoS One. 2026 Jan 21;21(1):e0327601. doi: 10.1371/journal.pone.0327601 (PMC12822936; doi:10.1371/journal.pone.0327601)
Supplement: Attachment — Submitted filename: Response to reviewers.docx [file pone.0327601.s010.docx]

**Response to Reviewers**
Manuscript ID: PONE-D-25-33165
Title: External validation of the Meggitt-Wagner, Texas University, SINBAD, and Saint Elian classifications for predicting major amputation in patients with diabetes at a public hospital in Peru

Dear Editor and Reviewers,

We are grateful for the constructive feedback and careful evaluation of our manuscript. We have revised the paper thoroughly to address all the comments provided by the reviewers and the academic editor. In the revised version, we have restructured specific sections of the discussion, clarified methodological details, improved the presentation of tables and figures, and expanded our interpretation of the findings in both clinical and public health contexts.

Below, we provide a detailed point-by-point response to each observation, indicating the changes made in the manuscript and justifying them where appropriate. All modifications have been incorporated into the revised version, which is submitted both with tracked changes and as a clean copy, as per journal requirements.

We thank the reviewers and the editorial team for their thoughtful comments, which have substantially improved the clarity and robustness of our work.

Sincerely,
Marlon A. Yovera-Aldana, MD, MSc

Reviewer #1:

1. Supplemental tables 1, 2, and 3 also show important classifications and indicators. These data should also be mentioned in the main text.

We appreciate the reviewer’s suggestion. In the original version, the main text reported only overall incidence, relative risks, and AUROC values, while the detailed statistical power, incidence by specific categories, and sensitivity/specificity parameters were presented exclusively in the supplementary tables. In the revised manuscript, we have incorporated the key findings from these tables into the Results section. Specifically, from S1 Table we added that the statistical power of the comparisons between curves exceeded 90% for SE versus MW and SINBAD, and was 78% for SE versus TU 3D. From S2 Table we included that most amputations occurred in MW grades 4–5 (92%), TU stage 3D (87%), SINBAD scores ≥5 (85%), and SE >20 (48%).

2. Clearly state the reasons and methods for integrating specific grades in the Meggitt-Wagner and Texas University classifications.

We thank the reviewer for this important observation. In the original version, the manuscript reported the results of the Meggitt-Wagner and Texas University classifications using integrated categories, but the rationale for grouping was not explicitly stated. In the revised version, we have clarified both the reasons and the methods used. Specifically, categories with very few or no cases of major amputation were collapsed with adjacent groups to ensure adequate statistical stability and valid estimations of risk. For the Meggitt-Wagner classification, grades 1–3 were combined because no amputations occurred in grades 1–2, and only three amputations were observed in grade 3. For the Texas University classification, most amputations were concentrated in stage 3D, while other stages had zero or very few events; therefore, categories were collapsed into “3D” versus “other stages” to allow meaningful risk estimates. These procedures were pre-specified to address sparse data issues and are commonly recommended in prognostic validation studies.

3. In order to provide practical suggestions for applying the research results to clinical practice, present specific examples of the classification scores applied in clinical settings in the conclusion section.

We appreciate this valuable suggestion. In the original version, the Conclusion highlighted the overall performance of the four classification systems but did not provide concrete examples for clinical use. We have revised the Conclusion to include specific thresholds that clinicians can apply at the bedside or in outpatient settings. For instance, we now indicate that patients with a Saint Elian score ≥18 or a Texas University stage 3D should be considered at high risk for major amputation and referred urgently to specialized multidisciplinary care. Similarly, patients with a Wagner grade ≥3 or a SINBAD score ≥5 should be closely monitored and prioritized for intensive management. These additions provide clear and practical guidance for clinical decision-making in resource-limited settings.

Reviewer #2:

Abstract:

4. Line 51, materials and methods: I would encourage the authors to provide a summary of the patient cohort as detailed in the body of the manuscript, specifically as it pertains to inclusion criteria and timeline.

**Original version (Abstract – Methods section):**
“A retrospective cohort study was conducted, including patients with a follow-up period of up to six months from hospital admission. The primary outcome was the occurrence of major amputation. For each classification system, the area under the receiver operating characteristic curve (AUROC) was calculated.”

**Revised version (Abstract – Methods section):**
“We conducted a retrospective cohort study at María Auxiliadora Hospital, one of the few referral centers in Lima with a specialized Diabetic Foot Unit. The study period was January 2015 to December 2019. Eligible patients had a lower-limb ulcer, complete clinical data recorded within 48 hours of the index healthcare encounter (hospital admission or outpatient evaluation), and at least six months of follow-up. Patients with venous ulcers or pressure ulcers related to immobilization were excluded.. The primary outcome was major amputation, defined as any procedure above the ankle. Prognostic performance of the four systems was assessed using the area under the receiver operating characteristic curve (AUROC), sensitivity, specificity, predictive values, and the Youden index

5. Line 57: “two thirds were male”: it may be best to put the absolute number of individuals and the corresponding proportion.

**Original version (Results – General characteristics):**
“The median age was 62 years (IQR: 54–69), and two thirds were male.”

**Revised version:**
“The median age was 62 years (IQR: 54–69), and 229 of 342 patients (67.0%) were male.”

6. Lines 65-67: “Strengthening the training of multidisciplinary teams in referral centers is essential to ensure the effective application of these classification systems in clinical decision-making.” It is difficult to see how this sentence naturally follows the first sentence of the conclusion that comments on the quality of the performance of the diabetic foot classification systems.

We have restructured the section to provide a logical flow from study findings to clinical and public health implications.

**Original version (Conclusion):**
“The Saint Elian and University of Texas classifications demonstrated good prognostic accuracy for major amputation at six months, while the Meggitt-Wagner and SINBAD classifications showed moderate performance. Strengthening the training of multidisciplinary teams in referral centers is essential to ensure the effective application of these classification systems in clinical decision-making.”

**Revised version (Conclusion):**
“The Saint Elian and University of Texas systems showed the best prognostic accuracy, while Meggitt-Wagner and SINBAD performed moderately. These findings provide clinicians with clear cut-off points to identify high-risk patients and support early interventions in referral settings. Their effective use depends on multidisciplinary teams trained in diabetic foot management, who can apply the classifications consistently and integrate them into decision-making. At the hospital and public health level, selecting the classification system best suited to available resources and patient profiles may optimize care pathways and contribute to reducing amputation rates..

Introduction:

7. Line 89: “In the local context” seems too broad. Without looking at the reference, it is difficult for the reader to determine whether the authors mean Peru as a whole, metropolitan Lima, or their hospital catchment area within metropolitan Lima.

We thank the reviewer for highlighting the ambiguity of the phrase “in the local context.” We agree that the scope needed to be specified in order to improve clarity for international readers.

**Original version(Introduction):**
“In the local context, diabetic foot affects approximately 30% of patients attending healthcare facilities [10]. However, only 11.5% to 15% of these patients achieve adequate metabolic control, defined as optimal levels of blood glucose, arterial pressure, and lipid profile [11,12]. Furthermore, diabetic foot is the reason for hospital admission in one out of every five patients with diabetes in Peruvian hospitals [13].”

**Revised version (Introduction):**

In the Peruvian context, diabetic foot affects approximately 5.9% of patients attending healthcare facilities [9]. It is also accounts one out of every five hospital admissions among patients with diabetes in Peruvian hospitals [10]. Limitations in healthcare personnel training, infrastructure, and funding hinder the establishment of multidisciplinary teams dedicated to diabetic foot care.[11] Furthermore, only 11.5% to 15% of these patients achieve adequate metabolic control —defined as optimal levels of blood glucose, blood pressure, and lipid profile —which further contributes to suboptimal clinical outcomes [12,13].

8. Line 96: “Therefore” implies a connection between the previous and current sentences, but such connection is difficult to make. Perhaps removing the word altogether would work best.

We thank the reviewer for noting the awkward transition at the beginning of this sentence. We agree that “Therefore” implies a causal link that is not clearly established and that rewrite the last paragraph to improve clarity and readability.

**Original version (Introduction):**
“Therefore, validating prognostic tools in settings different from those in which they were originally developed is essential to ensure their accuracy and applicability. Local contextual factors may influence the predictive performance of clinical scoring systems for major amputation.”

**Revised version (Introduction):**

Most clinical scoring systems for diabetic foot were developed and validated in high-income countries, and their predictive performance may not translate directly to Latin American settings. Differences in patient profiles, healthcare pathways, and resource availability can influence prognostic accuracy, underscoring the need for external validation. To address this gap, we compared the discriminative capacity of four widely used classifications—Meggitt-Wagner (MW), University of Texas score (TU), SINBAD, and Saint Elian (SE)—to predict major amputation at six months among patients managed at the Diabetic Foot Unit of a national referral

Methods:

9. As a whole, I think the methods require significant restructuring and additional clarifications.

For the restructuring: (1) the general order is mostly appropriate, with only a few exceptions, but that need to be corrected; (2) the content within the sections does not always fit with the stated title. There are also some redundancies.

For the additional clarifications, the most important ones in my opinion pertain to (1) data extraction: and (2) measurement: it is not clear which healthcare provider was responsible for registering patient data that are essential for calculation of the scores; outcome measurement: was ipsilaterality relative to the ulcer at presentation accounted for? Was the outcome possible to capture outside of their healthcare institution?

We thank the reviewer for this thorough assessment. In the revised manuscript, we have restructured the Methods section to improve coherence, reduce redundancies, and ensure that content is aligned with the appropriate subheadings. Regarding data extraction, we now specify that physicians from the Diabetic Foot Unit were responsible for systematically registering clinical, laboratory, and ulcer-related information at admission. For outcome measurement, we clarified that only major amputations ipsilateral to the presenting ulcer were considered, and ascertainment was based on hospital surgical records complemented by active follow-up. To minimize loss of outcome data, patients were contacted by telephone when follow-up visits were missed. We also added contextual information: very few hospitals in Lima have a specialized Diabetic Foot Unit, so most patients with new lesions preferentially attend our center, where they are known and managed as continuing patients, which ensures expedited care. Although Peru did not have an electronic medical record system at the time of the study and patient files were maintained manually, the probability of missing outcomes due to patients moving or seeking care elsewhere was considered low, but this limitation has been explicitly acknowledged

Full details:

10. Line 105: “Study design and clinical scenario”: the authors initiate a description of their healthcare institution regarding referral pathways and cases, then proceed further describe treatment protocol diabetic foot management in a completely another section (“Diagnostic and therapeutic algorithm at the Maria Auxiladora hospital” ) more than 80 lines later. For the benefit of the reader, it could be best to ensure all information about the hospital from which the data is sourced is mentioned at the same place in the body of the text.

In the revised manuscript, we consolidated the description of the hospital setting and care pathways into a single subsection at the beginning of the Methods, titled “Study setting and clinical management.” This subsection now integrates both the description of referral pathways, patient flow, and the specialized Diabetic Foot Unit, along with the diagnostic and therapeutic algorithm followed at our hospital.

11. Line 113: “Population, sample and sampling”: Where were suitable patients identified from? We come to understand later (e.g., “Preparation” section), that their institution has a diabetic foot database. Perhaps shifting the information from there would be appropriate. What were the patient’s reason for seeking medical care? It may simply be their foot ulceration, as lines 188-189 seem to indicate, but this should be explicitly clear.

In the revised manuscript, we now specify that suitable patients were identified from the diabetic foot database maintained by the Endocrinology Service at María Auxiliadora Hospital, which prospectively records all patients managed by the Diabetic Foot Unit. We also clarified that the primary reason for seeking medical care in all included cases was the presence of a lower-limb ulcer, infection, or ischemia, with most patients attending the Unit at the first sign of a lesion because of the hospital’s specialized referral role. This information has been relocated to the “Population and data source” subsection to ensure consistency and improve clarity.

12. Line 114 “The study include all patients with diabetic foot […]”: how was the diagnosis of diabetic foot made? A lower extremity ulceration and a concomitant diagnosis of diabetes? Exclusion criteria on lines 119-120 seem to suggest that greater refinement of possible, but irrelevant etiologies was performed. How were ulceration etiologies teased out?

In the revised version, we clarified that the diagnosis of diabetic foot was defined as the presence of a lower-extremity ulcer in a patient with a confirmed diagnosis of diabetes mellitus. The diagnosis of diabetes was based on medical history and laboratory records. For ulcer etiologies, differentiation was performed clinically by the Diabetic Foot Unit at the time of admission: venous ulcers were excluded based on typical location, chronic venous insufficiency signs, and absence of neuropathic or ischemic features; pressure ulcers were excluded if located on the heel and associated with prolonged immobilization or bedridden status. Only patients with neuropathic, ischemic, or mixed diabetic ulcers were included. This refinement has been explicitly described in the “Population and eligibility criteria” subsection of the Methods

13. Lines 116-118: “[…] patients also needed to have complete clinical information […] to allow for scoring […]”: what healthcare provider was tasked with extracting such clinical information? They mention the “Diabetic Foot Unit” on line 190, but whether physicians, nurses, or other healthcare professionals within that unit were responsible for this needs to clearer.

In the revised manuscript, we have clarified that the clinical information required for scoring was systematically extracted and registered by physicians from the Diabetic Foot Unit within 48 hours of admission. These physicians were endocrinologists or internal medicine specialists with specific training in diabetic foot management. Nursing staff supported data collection by recording wound care procedures and follow-up visits, but responsibility for the clinical and ulcer-related variables used in the classifications rested with the physicians. This has now been explicitly stated in the “Data extraction” subsection of the Methods.

14. Line 116: “[…] from the date of admission.”: this suggests inclusion of only inpatients, which is not the case in this study, as both in and outpatient health services were considered. using a better term to describe the index event is warranted. Perhaps "from the date of initiation of the index healthcare event" is more appropriate?

In the revised manuscript, we replaced this wording with: “…with a minimum follow-up of six months from the date of initiation of the index healthcare event (hospital admission or outpatient evaluation).”

15. Line 121: “All patients who met the eligibility criteria were included in the study”: this may not need to be mentioned, as it may go without saying.

We agree with the reviewer’s suggestion. In the revised manuscript, we removed the sentence “All patients who met the eligibility criteria were included in the study” to avoid redundancy, while retaining the explanation of sampling strategy and statistical power calculation.

16. Line 126: “Diabetic foot classifications”: this section would benefit from a more detailed rationale for selecting those specific classification schemes. Some of this rationale is detailed later, but is likely best placed in this section.

In the revised manuscript, we have added a detailed rationale for the selection of these four classifications in the “Diabetic foot classifications” subsection.

17. Line 160: “Major amputation”: (1) Was ipsilaterality relative to the ulcer at presentation a consideration? Although hard to know the exact proportion, there may be some individuals whose limb loss at 6 months was contralateral to the ulceration they initially presented with. This could significantly confound the results; (2) Where was this captured? At the index hospital only, or was it possible to identify amputation happening in another institution? This is crucial information, and if the authors could not collect data beyond their institution, this could represent a significant limitation to the study.

In the revised manuscript, we clarified that only amputations ipsilateral to the presenting ulcer were considered as outcomes; contralateral amputations within the follow-up period were not included in the analysis. We also specified that outcome ascertainment relied primarily on surgical records from the index hospital (María Auxiliadora Hospital). To minimize the risk of missing events, patients who did not return for scheduled follow-up visits were contacted by telephone, and their families were also approached when necessary. However, we acknowledge that amputations performed in other institutions may not have been fully captured, given the absence of a national electronic medical record system in Peru at the time of the study. We have explicitly mentioned this as a limitation in the revised manuscript.

18. Line 163: “Other variables”: was diabetes type a consideration?

In the revised manuscript, we clarified that all included patients had **type 2 diabetes mellitus.** This reflects the epidemiology of diabetes in Peru, which has one of the lowest prevalences of type 1 diabetes worldwide. Since no patients with type 1 diabetes were identified in the database, this variable was not included in the analysis. We added this clarification in the “Other variables” subsection of the Methods.

19. Line 176: “Ischemia”: the use of toe-brachial index or, to a lesser extent because of the confounding effect of medial calcinosis, ankle-brachial index is arguably better than qualitative assessment of arterial waveform for an accurate description of an individual’s degree of ischemia. This limitation was acknowledged in the paper discussion.

We thank the reviewer for the insightful suggestion regarding objective ischemia assessment. As noted, ankle-brachial index (ABI) and toe-brachial index (TBI) are more accurate than waveform analysis. However, practical limitations—especially in patients with extensive wounds, edema, or severe pain—often precluded ABI or TBI measurement in our context.

- Vriens B, D’Abate F, Ozdemir BA, Fenner C, Maynard W, Budge J, et al. Clinical examination and non-invasive screening tests in the diagnosis of peripheral artery disease in people with diabetes-related foot ulceration. Diabet Med. 2018;35: 895–902. doi:10.1111/DME.13634
- Criqui MH, Matsushita K, Aboyans V, Hess CN, Hicks CW, Kwan TW, et al. Lower Extremity Peripheral Artery Disease: Contemporary Epidemiology, Management Gaps, and Future Directions: A Scientific Statement From the American Heart Association. Circulation. 2021;144: e171. doi:10.1161/CIR.0000000000001005

20. Line 181: “Procedures”: one could argue the information in this section is best suited for an earlier mention in the methods section (perhaps within the “Study design and clinical scenario section”).

In response, we have consolidated all procedural information about hospital practice—including referral pathways, Diabetic Foot Unit evaluation, diagnostic-treatment algorithms, and data collection flow—into a single, early subsection titled “Study setting and clinical management.”

21. Lines 185-186: “Subsequently, the records were filtered and depurated according to predefined eligibility criteria for this study”: this statement appears somewhat intuitive. It may not be needed.

In the revised manuscript, we have removed this sentence.

22. Line 186: “depurated”: this term is not appropriate to describe data collection.

In the revised manuscript, we have removed this sentence.

23. Lines 201-203: the definition of ischemia is redundant at this point of the manuscript.

In the revised manuscript, we have removed the redundant restatement of the ischemia definition at this juncture.

24. Line 207: “Statistic analysis”: *Statistical

The section heading has been corrected to **“Statistical analysis”**

25. Lines 211-212: it is atypical to refer to the results of the study in the methods section of a manuscript. I would suggest they indicate that they have performed power calculation, then in the results section refer the reader to the corresponding supplemental table.

In the revised manuscript, we have separated methodology from results:

- **In Methods**, we retained the explanation that a power calculation was performed to ensure adequate sample size, but removed the numerical results.
- **In Results**, we now refer readers to the supplementary table containing the detailed power analysis, clearly labelled (S2 Table”).

Results

26. Although perhaps not possible given the smaller cohort size, stratifying analyses based on admission status would probably be important. Requiring admission most likely indicate more severe disease and increase the risk of limb loss considerably.

We thank the reviewer for this insightful observation. In the revised analysis, we stratified discriminative performance according to admission status. As expected, patients requiring hospitalization showed lower predictive accuracy across all scales, reflecting their more severe clinical profile. For example, the Saint Elian classification decreased from excellent discrimination in the overall cohort (AUROC 0.90, 95% CI 0.86–0.93) to moderate discrimination among hospitalized patients (AUROC 0.74, 95% CI 0.65–0.82). Similarly, Meggitt–Wagner declined from good discrimination overall (AUROC 0.80, 95% CI 0.76–0.84) to poor performance in inpatients (AUROC 0.60, 95% CI 0.50–0.69). SINBAD also fell from moderate to very poor discrimination (AUROC 0.75 vs. 0.58). These findings confirm the reviewer’s point that hospitalization status reflects a sicker population in whom outcome prediction is more challenging.

Importantly, it was not possible to estimate discriminative capacity among outpatients alone, since no major amputations were recorded in this subgroup during follow-up. We have clarified this explicitly in the Results and Discussion sections.

27. Line 238: general characteristics: a statement about the total number of patients would help the reader get a better idea of size of the patient cohort.

We agree and have added an explicit statement at the start of the Results section. The first sentence now reads:

“A total of 342 patients with diabetic foot ulcers were included in the study.”

28. Line 293: Table 3 has a typo in the first row, third column (“¿”)

We removed the stray “¿” symbol; the cell now displays the correct label/value consistent with the column header

Discussion

29. The discussion needs re-structuring. Although most of the essential information is present, it seems to be spread out in different sections, sometimes seemingly inappropriately. Furthermore, some of the cited sources ([30]) do not appear in the bibliography. I would encourage the authors to double check the references to ensure they are accurate.

All references were verified for accuracy in numbering, format, and presence in the reference list.

Full details: (note that line numbers appear to be missing from now on)

30. “Main findings”: terms like “good” and “moderate” are used to describe the model discriminative ability: what cutoffs were used to make such statements should be made explicit in the methods section.

To facilitate interpretation of prognostic accuracy, we prespecified thresholds for the area under the ROC curve. An AUROC of 0.50 indicates chance-level discrimination; 0.51–0.59, very poor discrimination; 0.60–0.69, poor discrimination; 0.70–0.79, moderate discrimination; 0.80–0.89, good discrimination; and ≥0.90, excellent discrimination. These thresholds were applied consistently when describing the performance of the models in the Discussion.

Schober P, Mascha EJ, Vetter TR. Statistics from A (Agreement) to Z (z Score): A Guide to Interpreting Common Measures of Association, Agreement, Diagnostic Accuracy, Effect Size, Heterogeneity, and Reliability in Medical Research. Anesth Analg. 2021;133: 1633–1641. doi:10.1213/ANE.0000000000005773

31. “Theoretical and practical implications”: (1) I would encourage the authors how this could change practice within their hospital. Perhaps adopting one of the better performing models to screen for high risk individuals that require intensified surveillance is in the cards? Do their findings have more broad implications. (2) The section’s last paragraph would be best placed in their section about previous studies.

We thank the reviewer for these valuable suggestions.

“At the hospital level, adopting Saint Elian or the University of Texas system as primary screening tools could help clinicians promptly identify patients at highest risk for major amputation, enabling intensified surveillance, targeted vascular evaluation, and timely multidisciplinary interventions. Wagner at grade ≥3 may function as a high-sensitivity rule-out threshold at first contact, while SINBAD at score ≥5 can facilitate communication and auditing across services. Other scores such as WIfI (Wound, Ischemia, and foot Infection) could not be evaluated in this cohort because they require systolic pressure measurements (ankle–brachial index, toe–brachial index, or transcutaneous oxygen), which were not systematically available; this highlights the importance of aligning tool selection with feasible diagnostic practices[34]. Finally, training and standardized application are essential to limit interobserver variability and ensure reproducible, clinically meaningful use of any classification system..”

The last paragraph was moved in section about previous studies.

32. “Public health importance”: (1) line 5: “World guidelines” is very broad. I would encourage the author to explicitly state which guidelines they're referring to. Furthermore, the source is a cohort study, rather than any guidelines. The authors should cite the guidelines directly. (2) last line: source 30 is not mentioned in the manuscript references.

In the revised manuscript, we replaced the vague phrase “World guidelines” with a precise reference to the 2019 International Working Group on the Diabetic Foot (IWGDF) guidelines on ulcer classification . The corrected text now reads:

“At the primary level, the Peruvian diabetic foot guidelines consider only the Wagner classification for reference purposes [29]. The International Working Group on the Diabetic Foot (IWGDF 2023) guidelines recommend the use of SINBAD because it is simple and easy to use, and could be an alternative to improve communication and benchmarking across institutions.”

Additionally, we corrected all reference list.

33. “Strength and limitations”: lines 2-3 from the bottom: “doctors of the endocrinology service carried out an active search for the outcomes”: whether they were able to accurately extract outcome occurrence in a healthcare institution other than theirs will be very important information and, as detailed above, will need to be described in the methods of the paper.

In the revised manuscript, we clarified that outcome ascertainment was based primarily on surgical records at María Auxiliadora Hospital, complemented by active follow-up conducted by endocrinology service physicians during hospitalization and after discharge. For patients who missed follow-up visits, outcomes were verified by telephone contact with patients or their relatives. However, we acknowledge that amputations performed in other healthcare institutions could not always be captured, given the absence of a national electronic medical record system in Peru at the time of the study. This clarification has now been included both in the **Methods (Outcome ascertainment)** section and explicitly noted as a **limitation** in the Strengths and limitations section.
