# Supplementary material for: External validation of the Meggitt-Wagner, Texas University, SINBAD, and Saint Elian classifications for predicting major amputation in patients with diabetes at a public hospital in Peru
Source: PLoS One. 2026 Jan 21;21(1):e0327601. doi: 10.1371/journal.pone.0327601 (PMC12822936; doi:10.1371/journal.pone.0327601)
Supplement: Attachment — Submitted filename: PointByPointResponse_rev 2.docx [file pone.0327601.s011.docx]

Editor-in-Chief,

We thank the Editor and reviewers for the time dedicated to evaluating our manuscript and for the constructive feedback provided. We appreciate the positive comments and have revised the manuscript accordingly, incorporating additional analyses, clarifying methods, and improving the overall presentation.

A detailed point-by-point response is provided below, and all modifications in the manuscript are highlighted in red. We trust that the revised version satisfactorily addresses all comments.

Sincerely,

Marlon Yovera-Aldana, on behalf of all co-authors

**Reviewer #3**

**Comment 1**: *"* *Please consider reporting using Strobe Guidelines as many important elements are missing. Alternatively for clinical prediction, you can use Tripod Reporting guidelines "*

**Response 1:** We thank the reviewer for this observation. We have ensured full adherence to the STROBE guidelines for cohort studies and the TRIPOD statement for external validation studies. A dedicated “Reporting guidelines” subsection has been added in the Methods section, and the completed STROBE and TRIPOD checklists have been included as Supplementary Tables S1 and S2. In addition, several reporting elements required by these guidelines have been completed: (1) missing data proportions are now explicitly reported and summarized in a supplementary table; (2) the direction and potential magnitude of key limitations are now described; and (3) the study design section has been expanded to provide clearer methodological detail.

**Comment 2**: *“What % of data was missing and how was missing data being managed?”*

**Response 2:** We thank the reviewer for this comment. The proportion of missing data for each variable has been reported and summarized in Supplementary Table S3. Missingness ranged from 0% to 63.7% across variables. As detailed in the Methods section, no imputation procedures were applied; all analyses were performed using complete-case data for each variable. In addition, 15 patients were excluded because they had incomplete information regarding the outcome or one or more of the variables required to compute the diabetic foot classification scales (Figure 1).

**Comment 3**: *“What about minor amputations? Why were they excluded?”.*

**Response 3:** We thank the reviewer for this comment. The study was designed to evaluate the prognostic performance of the four diabetic-foot classification systems for major amputation, which was the predefined primary outcome. For this reason, minor amputations were not included in the primary analyses. The definition of the outcome has been clarified in the Methods section to specify that the “no major amputation” group includes both patients with no amputation and those who underwent minor amputation. The proportion of minor amputation has also been added to Figure 1 and Table 1.

Because different studies evaluate either major amputation or amputation at any level, we performed a complementary analysis using the broader outcome “any amputation” (major or minor combined). As this was not the primary objective of the study, the complete results of this complementary analysis are presented in Supplementary Table S7.
